# A successfully treated case of Lemmel syndrome with pancreaticobiliary maljunction: A case report

**DOI:** 10.1016/j.ijscr.2020.06.080

**Published:** 2020-06-24

**Authors:** Mitsuru Yanagaki, Hiroaki Shiba, Shin Hagiwara, Masato Hoshino, Hitoshi Sakuda, Yoshiyuki Furukawa, Katsuhiko Yanaga

**Affiliations:** a Department of Digestive Surgery, AOI Universal Hospital, 2-9-1, Tamachi, Kawasaki-ku, Kawasaki-city, Kanagawa, 210-0822, Japan; b Department of Hepato-Biliary-pancreatic Surgery, Jikei University School of Medicine, 3-25-8 Nishi-shinbashi, Minato-ku, Tokyo, 105-8461, Japan; c Department of Cardiovascular Surgery, AOI Universal Hospital, 2-9-1, Tamachi, Kawasaki-ku, Kawasaki-city, Kanagawa, 210-0822, Japan

**Keywords:** ERCP, endoscopic retrograde cholangio-pancreatography, PAD, periampullary diverticula, AST, serum aspartate aminotransferase, ALT, serum alanine transaminase, CT, computed tomography, CBD, common bile duct, NPO, nothing per os, MRCP, magnetic resonance cholangiopancreatography, Lemmel syndrome, Pancreaticobiliary maljunction, Parapapillary diverticulum

## Abstract

**Background:**

Lemmel syndrome is a rare condition that leads to cholangitis and/or pancreatitis due to intraduodenal diverticulum. Surgery is considered for the treatment of severe or repeated symptoms in patients with this condition.

**Case presentation:**

An 81-year-old woman was admitted to our hospital, complaining of general fatigue, BT 38.8 degree, and right hypochondoralgia. Her hepatobiliary enzyme levels were elevated, and enhanced abdominal computed tomography revealed dilation of the common bile duct and intraduodenal diverticulum. After restarting oral intake, her symptoms were exacerbated. Endoscopic retrograde cholangio-pancreatography (ERCP) revealed pancreaticobiliary maljunction and parapapillary diverticulum. Under a diagnosis of Lemmel syndrome with pancreaticobiliary maljunction complicated by acute pancreatitis and cholangitis, we performed extrahepatic bile duct resection with cholecystectomy and papilloplasty. Her postoperative course was uneventful, and the patient was discharged 20 days after surgery. She remains well at 5 months after surgery.

**Conclusion:**

We herein report a successfully diagnosed and treated case of Lemmel syndrome with pancreaticobiliary maljunction.

## Highlights

• Lemmel syndrome is a rare condition that leads to cholangitis and/or pancreatitis due to intraduodenal diverticulum.
• Surgery is considered for the treatment of severe or repeated symptoms in patients with this condition.
• We herein report a successfully diagnosed and treated case of Lemmel syndrome with pancreaticobiliary maljunction.

## 1 Introduction

Duodenal diverticula are most often found in the second portion of the duodenum adjacent to the ampulla of Vater. These diverticula are pseudo-diverticula consisting of outpouchings of the mucosa, which lack the muscular layer. When these diverticula are located within 2–3 cm of the ampulla of Vater, they are termed periampullary diverticula (PAD) [1]. PAD is usually asymptomatic; however, in rare instances, diverticular inflammation leads to pancreaticobiliary complications [2]. Obstructive jaundice can develop secondary to periampullary diverticula without choledocholithiasis in the setting of Lemmel syndrome [3]. We present a case of Lemmel syndrome with pancreaticobiliary maljunction. Informed consent was obtained from the patient for the publication of this report. This work is reported in line with the SCARE criteria [4] for case report publication.

## 2 Case presentation

**Fig. 1:**
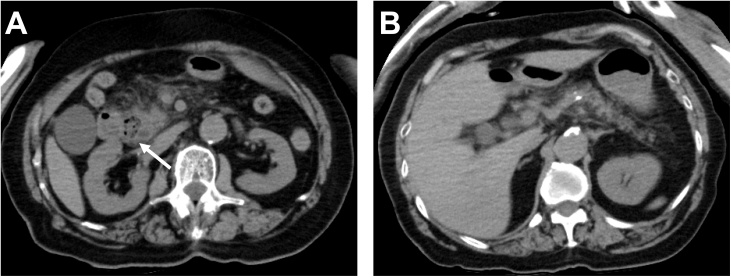
Plain axial CT of the abdomen and pelvis. Plain axial CT of the abdomen and pelvis demonstrated (A) inflammatory changes around the pancreatic head and large periampullary diverticulum (arrowhead). (B) A coronal slice showing no inflammatory changes around the pancreatic body and tail.

**Fig. 2:**
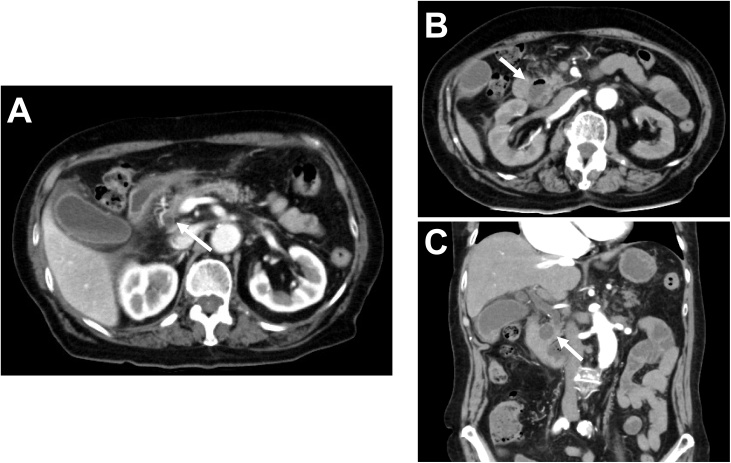
Contrast-enhanced axial and coronal CT of the abdomen and pelvis. Contrast-enhanced CT demonstrated (A) slight dilation of the CBD (arrowhead). (B) A coronal slice showing a large periampullary diverticulum containing fluid with wall thickening, mucosal enhancement, and surrounding inflammatory changes consistent with duodenal diverticulitis (arrowhead).

**Fig. 3:**
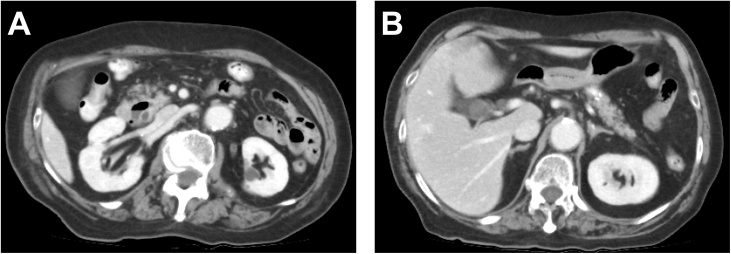
Contrast-enhanced axial CT of the abdomen and pelvis. Contrast-enhanced CT demonstrated a significant improvement of the inflammation in the pancreatic head (A) and body and tail of the pancreas(B).

**Fig. 4:**
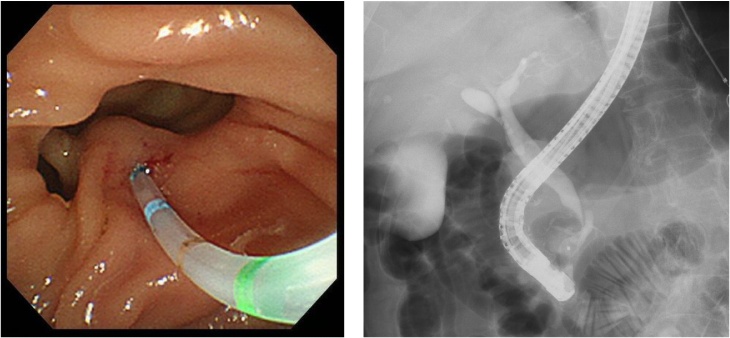
ERCP. ERCP revealed pancreaticobiliary maljunction and a periampullary diverticulum.

**Fig. 5:**
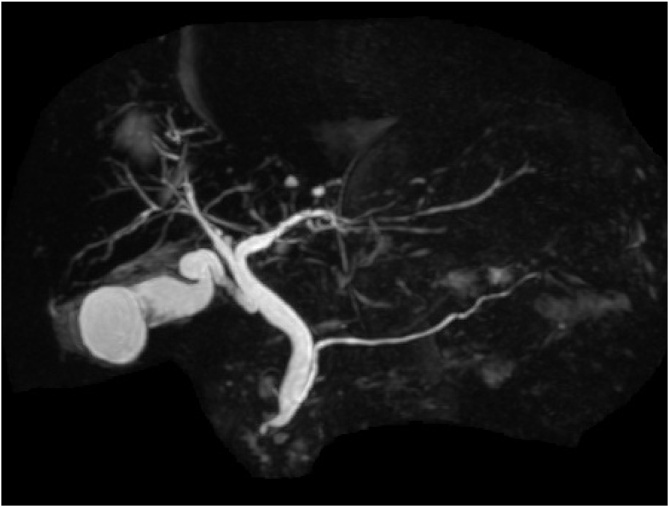
MRCP. ERCP revealed pancreaticobiliary maljunction.

An 81-year-old previously healthy woman presented to the emergency department of our hospital without any significant past medical history. She had general fatigue, BT 38.8 degree, and right hypochondoralgia, and was admitted to our hospital. The patient denied any nausea, vomiting, melena, hematochezia, or hematemesis. A physical examination revealed no particular findings; jaundice was not observed. A laboratory analysis revealed that her hemoglobin and bilirubin metabolites were normal, but her white cell count (<9300) was 27,000/μl, and her CRP level (<0.3) was 8.87 mg/dl. Furthermore, her serum aspartate aminotransferase (AST < 40) was 337 U/L, her alanine transaminase (ALT < 45) level was 143 U/L, her amylase level (<129) was 1100 U/L, and her total bilirubin level (<1.1) was 1.0 mg/dl. Plain computed tomography of the abdomen and pelvis revealed a periampullary duodenal diverticulum with surrounding inflammatory changes, and we diagnosed acute pancreatitis and cholangitis (Fig. 1). The next day, contrast-enhanced computed tomography (CT) of the abdomen and pelvis revealed slight dilation of the common bile duct (CBD) and a huge periampullary diverticulum containing fluid with wall thickening, mucosal enhancement and surrounding inflammatory changes consistent with duodenal diverticulitis (Fig. 2). She was diagnosed as pancreatitis quantifying a severity score of “low grade pancreatitis” according to diagnosis criteria; Tokyo Guideline 2018 [5] and cholangitis quantifying a severity score of “low grade cholangitis” according to diagnosis criteria; Revision of the Atlanta classiﬁcation and deﬁnitions [6]. The patient was subsequently classified as nil per os (NPO). She completed a 12-day course of intravenous antibiotics with ampicillin and sulbactam and her symptom, inflammatory reaction and liver function were gradually improved. One week later, CT revealed a significant improvement of the inflammation (Fig. 3). After restarting oral intake, her symptoms were exacerbated. Endoscopic retrograde cholangio-pancreatography (ERCP; Fig. 4) and magnetic resonance cholangiopancreatography (MRCP; Fig. 5) revealed pancreaticobiliary maljunction and a periampullary diverticulum. Under a diagnosis of Lemmel syndrome with pancreaticobiliary maljunction complicated by acute pancreatitis and cholangitis, we performed extrahepatic bile duct resection with cholecystectomy, hepaticojejunostomy, resection of the diverticulum and papilloplasty. The operation time was 230 min, and the estimated blood loss was 200 ml. Histology revealed no atypical cells in the gallbladder and the CBD. The patient’s postoperative course was uneventful and was discharged 20 days after surgery. She remains well at 5 months after surgery.

## 3 Discussion

Primary or true duodenal diverticula represent mucosal outpouchings devoid of muscle layer. Duodenal diverticula are classified based on their location. Among the different types of duodenal diverticula, periampullary duodenal diverticula are the most common [7]. The majority of periampullary diverticula are asymptomatic; however, biliopancreatic complications such as recurrent biliary calculi, obstructive jaundice, cholangitis, acute or chronic pancreatitis can result from mechanical compression by a large, distended due to poorly emptying diverticulum or due to motility dysfunction of the sphincter of Oddi, reflux of intestinal content into the ducts and bacterial overgrowth [8]. Complications related to inflammation such as diverticulitis, hemorrhage, perforation or fistula formation may also occur [1,[9], [10], [11], [12]]. These complications can result from mechanical compression by a large diverticulum that is distended due to poor emptying or due to motility dysfunction of the sphincter of Oddi, reflux of the intestinal content into the ducts and bacterial overgrowth [13,14].

Lemmel syndrome was ﬁrst described in 1934 by Lemmel as obstructive jaundice caused by periampullary duodenal diverticulum in the absence of choledocholithiasis or neoplasm [7]. Imaging studies, such as CT, MRCP, and barium studies, are essential for diagnosing this condition. On CT and MRCP, PAD may appear as thin-walled cavitary lesions on the wall of the second portion of the duodenum. Barium studies show the PAD with contrast-ﬁlled outpouching that arises from the wall of the descending duodenum [7]. Sometimes these diverticula can be filled with fluid and misdiagnosed as a pancreatic abscess, cystic neoplasm in the pancreatic head, or metastatic lymph node [15]. In cases of biliary obstruction, excision of the diverticulum is an appropriate procedure; however, this can be difficult and is associated with significant mortality and morbidity [16]. Endoscopic sphincterotomy or stenting are reasonable alternatives in high-risk patients [[17], [18], [19]], although the failure and complication rates are considerable as the papilla is most often located in or adjacent to the diverticulum [20]. In this case, we incised the anterior wall of the papilla for 2 cm and approximated its mucosal surfaces with non-absorbable suture as papilloplasty for chronic pancreatitis prevention [21].

## 4 Conclusions

We reported a successfully treated case of Lemmel syndrome with pancreaticobiliary maljunction by extrahepatic bile duct resection with cholecystectomy, hepaticojejunostomy, and papilloplasty.

## Conflicts of interest

Not applicable.

## Sources of funding

Not applicable.

## Ethical approval

Not applicable

## Consent

Not applicable.

## Author contribution

MY, HS, SH, MH, HS, YF, and KY designed the research, and MY and HS wrote the paper. All authors read and approved the final manuscript.

## Registration of research studies

Our report is not research study. It is a case report.

## Guarantor

Mtsuru Yanagaki.

## Provenance and peer review

Not commissioned, externally peer-reviewed.

## References

[1] Zoepf T., Zoepf D.S., Arnold J.C., Benz C., Riemann J.F. (2001). The relationship between juxtapapillary duodenal diverticula and disorders of the biliopancreatic system: analysis of 350 patients. Gastrointest. Endosc..

[2] Gore R.M., Ghahremani G.G., Kirsch M.D., Nemcek A.A., Karoll M.P. (1991). Diverticulitis of the duodenum: clinical and radiological manifestations of seven cases. Am. J. Gastroenterol..

[3] Lemmel G. (1934). Die klinische bedeutung der duodenaldivertikel. Arch Digest. Dis..

[4] Agha R., Borrelli M., Farwana R., Koshy K., Fowler A., Orgill D. (2018). For the SCARE GroupThe SCARE 2018 statement: updating consensus Surgical CAse REport (SCARE) guideline. Int. J. Surg..

[5] Mayumi T., Okamoto K., Takada T., Strasberg S.M., Solomkin J.S. (2018). Tokyo Guidelines 2018: management bundles for acute cholangitis and cholecystitis. J. Hepatobiliary. Sci..

[6] Banks P.A., Bollen T.L., Dervenis C. (2013). Classiﬁcation of acute pancreatitis-2012: revision of the Atlanta classiﬁcation and deﬁnitions by international consensus. Gut.

[7] Desai K., Wermers J.D., Beteselassie N. (2017). Lemmel syndrome secondary to duodenal diverticulitis: a case report. Cureus.

[8] Desai Keyur, Wermers Joshua D., Beteselassie Nebiyu (2017). Lemmel syndrome secondary to duodenal diverticulitis: a case report. Cureus.

[9] Lobo D.N., Balfour T.W., Iftikhar S.Y., Rowlands B.J. (1999). Periampullary diverticula and pancreaticobiliary disease. Br. J. Surg..

[10] Wu S.D., Su Y., Fan Y., Zhang Z.H., Wang H.L., Kong J., Tian Y. (2007). Relationship between intraduodenal peri-ampullary diverticulum and biliary disease in 178 patients undergoing ERCP. HBPD INT.

[11] Gudjonsson H., Gamelli R.L., Kaye M.D. (1988). Symptomatic biliary obstruction associated with juxtapapillary duodenal diverticulum. Dig. Dis. Sci..

[12] Egawa N., Anjiki H., Takuma K., Kamisawa T. (2010). Juxtapapillary duodenal diverticula and pancreatobiliary disease. Dig. Surg..

[13] Vitturi N., Simoni F., De Stefano F., Orlando R., Lirussi F., Realdi G. (2010). Paravaterian diverticula presenting as acute cholangitis in two very elderly patients. J. Gastrointestin. Liver Dis..

[14] san Román A.L., Moreira V.F., García M., Merono E., Boixeda D. (1994). Direct compression by a duodenal diverticulum causing common bile duct obstruction. Endoscopy.

[15] Rouet J., Gaujoux S., Ronot M., Palazzo M., Cauchy F., Vilgrain V., Belghiti J., O’Toole D., Sauvanet A. (2012). Lemmel’s syndrome as a rare cause of obstructive jaundice. Clin. Res. Hepatol. Gastroenterol..

[16] Yoneyama F., Miyata K., Ohta H., Takeuchi E., Yamada T., Kobayashi Y. (2004). Excision of a juxtapapillary duodenal diverticulum causing biliary obstruction: report of three cases. J. Hepatobiliary Surg..

[17] Thomas E., Reddy K.R. (1982). Cholangitis and pancreatitis due to juxtapapillary duodenal diverticulum. Endoscopic sphincterotomy is the other alternative in selected cases. Am. J. Gastroenterol..

[18] Buse P.E., Edmundowicz S.A. (1991). Proximal common bile duct obstruction secondary to a periampullary duodenal diverticulum: successful treatment with endoscopic stenting. Gastrointest. Endosc..

[19] Chiang T.H., Lee Y.C., Chiu H.M., Huang S.P., Lin J.T., HP Wang (2006). Endoscopic therapeutics for patients with cholangitis caused by the juxtapapillary duodenal diverticulum. Hepatogastroenterology.

[20] Tyagi P., Sharma P., Sharma B.C., AS Puri (2009). Periampullary diverticula and technical success of endoscopic retrograde cholangiopancreatography. Surg. Endosc..

[21] Nardi G.L. (1960). Technique of sphincteroplasty in recurrent pancreatitis. Surg. Gynecol. Obstet..

